# Predicting risk of hospital and emergency department use for home care elderly persons through a secondary analysis of cross-national data

**DOI:** 10.1186/s12913-014-0519-z

**Published:** 2014-11-14

**Authors:** John N Morris, Elizabeth P Howard, Knight Steel, Robert Schreiber, Brant E Fries, Lewis A Lipsitz, Beryl Goldman

**Affiliations:** Institute for Aging Research, Hebrew SeniorLife, 1200 Centre Street, Boston, MA 02131 USA; Northeastern University, School of Nursing, 360 Huntington Avenue, Boston, MA 02115 USA; Hackensack University Medical Center, 30 Prospect Avenue, Hackensack, NJ 07601 USA; Institute of Gerontology and Geriatric Research, Education and Clinical Center, University of Michigan, Ann Arbor VA Healthcare Center, 300 NIB, 933 NW, Ann Arbor, MI 48109 USA; Kendal Outreach LLC, 1107 E Baltimore Pike, Kennett Square, PA 19348 USA

## Abstract

**Background:**

Older adults remain the highest utilization group with unplanned visits to emergency departments and hospital admissions. Many have considered what leads to this high utilization and the answers provided have depended upon the independent measures available in the datasets used. This project was designed to further understanding of the reasons for older adult ED visits and admissions to acute care hospitals.

**Methods:**

A secondary analysis of data from a cross-national sample of community residing elderly, 60 years of age or older, and most of whom received services from a local home-care program was conducted. The assessment instrument used in this study is the interRAI HC (home care), designed for use in assessing elderly home care recipients. The model specification stage of the study identified the baseline independent variables that do and do not predict the follow-up measure of hospitalization and ED use. Stepwise logistic regression was used next to identify characteristics that best identified elders who subsequently entered a hospital or visited an ED. The items generated from the final multivariate logistic equation using the interRAI home care measures comprise the interRAI Hospital-ED Risk Index.

**Results:**

Independent measures in three key domains of clinical complications, disease diagnoses and specialized treatments were related to subsequent hospitalization or ED use. Among the eighteen clinical complication measures with higher, meaningful odds ratios are pneumonia, urinary tract infection, fever, chest pain, diarrhea, unintended weight loss, a variety of skin conditions, and subject self-reported poor health. Disease diagnoses with a meaningful relationship with hospital/ED use include coronary artery disease, congestive heart failure, cancer, emphysema and renal failure. Specialized treatments with the highest odds ratios were blood transfusion, IV infusion, wound treatment, radiation and dialysis. Two measures, Alzheimer’s disease and day care appear to have a protective effect for hospitalization/ED use with lower odds ratios.

**Conclusions:**

Examination into “preventable” hospitalizations and re-hospitalizations for older adults who have the highest rates of utilization are occurring beneath an umbrella of assuring the highest quality of care and controlling costs. The interRAI Hospitalization-ED Risk Index offers an effective approach to predicting hospitalization utilization among community dwelling older adults.

## Background

Reducing the rate at which elders make visits to emergency departments (ED) or are admitted to an acute hospital is a priority around the globe [1-4]. In the United States the issue goes back several decades as the Federal government introduced a diagnostic-based prospective payment system to incent hospitals to reduce the average length of stay for Medicare recipients. In 1980, the in-hospital average stay for elders was 10.7 days, while 29 years later it had decreased to only 5.5 days [5]. With the 2010 rollout of the Patient Protection and Affordable Care Act, the US government focused next on decreasing the occurrence of what are called preventable readmissions. In 2013 the Medicare Pay-for Reporting Program specifically targeted hospitals with “higher than expected” readmission rates for patients treated for heart failure, myocardial infection or pneumonia [6].

At the same time the number of visits to emergency departments has continued to grow. This is all the more striking as non-hospital based surgical centers [7] and a variety of innovative community care programs have become widespread [8-10]. A 2009 study reported that for every 100 elders in the United States, 52.2 emergency room visits were made annually resulting in a total of 19,818,000 visits during the year [11]. Using search engines available at a large US university library in the US, the authors reviewed the existing literature within the last 5 years using key terms including older adult hospitalization, emergency room use, disease and disabilities requiring visits to hospitals and emergency rooms. Subsequently, a review of papers published on quantitative methods that examined the relationship between hospitalization/emergency room visits and designated qualities or states of older adults was completed and summarized.

Age is unquestionably related to one’s risk of hospital use. In 2012, 13.6% of persons 65-74 had one or more hospitalizations, with the rate rising to 20.8% for persons 85 years of age or older [12]. The effect of an aging baby boom generation will undoubtedly be profound [13].

What other than age do we know about the factors driving this use? In a longitudinal cohort study of older adults with dementia, the Ambulatory Care Sensitive Conditions study, three conditions -- bacterial pneumonia, congestive heart failure and a urinary tract infection -- accounted for 2/3 of all potentially preventable hospital admissions. For certain sub-groups (e.g., persons with advanced Alzheimer’s disease) functional parameters have been shown to be a factor in determining the use of emergency department and acute hospital care [14,15]. Other studies have identified simple risk measures such as a person’s self-report of poor health, as well as more complex interrelated measures that apply as the elder is nearing the end of life (e.g., weight loss, fever, and skin breakdown in an individual with a diagnosis of cancer) [15]. The Probability of Repeated Admission index (PRA), an eight item self-administered questionnaire used prospectively in three European countries, demonstrated that high risk elders were 2.3 times more likely to have a hospital admission [16]. In one study, four of the noted criteria included low BMI, low physical activity and a measure of satisfaction with muscle strength and endurance, elders with three or four of these criteria being more often hospitalized and having longer hospital stays [17]. In another study the number of symptoms but not specific symptoms was associated with increased hospitalization and ED visits [18].

Poly-pharmacy has also been identified as an important factor in a number of studies [19] and the use of potentially inappropriate medications as well as overuse or underuse of medications was associated with a higher risk of hospitalization [20]. Examination of community dwelling older adults in Florence Italy revealed that both taking five or more medications and having a prior hospitalization were significant predictors of hospital admissions [21]. A Finnish study examined the association between the risk of hospitalization and the Drug Burden Index (a measure of exposure to anticholinergic and sedative medications). Higher index scores were associated with a higher rate of hospitalization in elders [22].

Finally, recent hospitalization has also been associated with both ED use and re-hospitalization [23-26]. Currently, there is pressure to better understand the re-hospitalization phenomenon. Penalties from CMS in the United States are now in effect for several diagnostic groups, but whether this strategy will work or whether certain classes of hospitals will be penalized is open to debate. Furthermore, as demonstrated by the Dartmouth Atlas, hospital utilization rates vary 2-3 fold across the United States [27]. There are local resource factors at play including the supply of hospital beds for some diseases, local practice patterns and differences in clinical decision-making.

The research described in this paper is designed to further our understanding of the complex set of factors that are related to an older adult’s ED visits and admissions to acute care hospitals. This paper’s particular strengths rest first on is its use of a complex set of predictive measures as derived from an internationally used assessment system – the interRAI Home Care tool; and second, on our ability to evaluate hospital and ED use for older adults in governmentally-sponsored home care programs in several US states, several Canadian Provinces, and one European country. All study subjects are clients of the formal home care programs in their jurisdiction and they were assessed using the same set of measures as derived from the widely used interRAI Home Care tool [28]. As a first step in this research, the many available independent measures in the interRAI-HC were reviewed to identify the baseline clinical, diagnostic, treatment and other factors that are and are not related to subsequent hospital use or emergency department visits. Next, using a multivariate logistic analytic strategy the baseline set of identified independent measures are reduced into a more parsimonious set of predictors. Through this reduction we can better understand the key factors that are correlated with such use. Finally, the set of covariates from the logistic runs are summarized within a categorical risk model, the Hospital-ED Risk Index. The strength of the index rests on the breath of risk factors that emerge from the extensive set of independent measures tested. Finally, we display hospital and ED rates for the categories in total by dataset and by a set of selected disease categories.

## Methods

### Sample and data

The risk relationships tested are based on secondary analyses of data from a cross-national sample of elderly home care clients, all of whom are 60 years of age or older. Prior to initiating the analysis we ask whether the same general risk forces are at play in each of the data cohorts, a requirement for us to pool the data for the subsequent risk analyses. The dataset, maintained by interRAI (an international not-for-profit consortium) consists of computerized home care records provided by governmental agencies. The case files include all persons in each jurisdiction who are receiving government-supported home care services. The interRAI collaborative network includes researchers in 33 countries. This consortium collects and interprets high-quality data about characteristics and outcomes of persons served. From Finland, the available data include city wide home care cohorts from much of the country (note, in Finland the city is the organizational home-care service delivery structure). In Canada, the data represent all home care clients receiving government-supported services in the Provinces of Ontario and Manitoba. The home care data in the United States represent all state supported home care clients in Massachusetts, Michigan and Georgia.

Ethics approval of this project was provided by the internal review board of Hebrew SeniorLife., allowing the use of de-identified data from the interRAI repository for research purposes. In total, baseline interRAI assessments are available for 585,888 elderly home care clients, while follow-up data (on average about six-months after baseline) are available for 316,934 persons. In the analytic work for this paper two-thirds of the available cases were randomly identified for model derivation (n = 390,356), one-third were reserved for model re-test validation (n = 195,532).

All personal identifiers were removed from the database, leaving only a code representing the source of the data (e.g., home cares sites in the Province of Ontario). All assessments were performed by assessors, trained in the use of the assessment instrument. The training occurred separately in each country (state or province), but in each instance followed models specified by interRAI [29,30]. Therefore, the reliability of the available data elements should be excellent and consistent with those reported previously [30-32].

The assessment instrument used in this study is the interRAI HC (home care). This tool is one of a suite of assessment instruments developed by the international interRAI consortium. The tool consists of more than 300 items. and was designed for use in assessing elderly home care recipients, providing measures relevant to care planning, resource allocation, outcome measurements and quality assessment. As such, the item set is quite comprehensive. The demographics component includes age, gender, race, marital status, and living arrangement. Cognitive measures include memory, executive function, delirium, communication skills, and depression. Functional measures include activities of daily living (ADLs) and instrumental activities of daily living (IADLs). Disease diagnoses, as reported by the clinical assessors, include both an extensive list of specific conditions (for which a forced answer of present or not present was required) with items that specify diseases of the heart, circulation, neurological and muscle-skeletal systems, and senses, as well as infection and other conditions. There is also a set of open-ended ICD disease classifications. The available list of acute conditions is quite extensive and includes diarrhea, fever, vomiting, chest pain, pain frequency and intensity, shortness of breath, edema, dizziness, unsteady gait, delusions, hallucinations, falls, weight loss, pressure ulcers, stasis ulcers, skin tears and abrasions as well as surgical wounds, and chewing problems. Finally, there are measures of drug use, treatments, service use, estimated proximity to death, self-reported health status, and the degree of medical instability.

The interRAI assessment instruments contain two assessor-based (not record review based) health care utilization items on which the dependent measure was based. One provides a count of the number of times in the 90 days prior to the assessment that the person was admitted to a hospital with an overnight stay. A second item provides a count of the number of ED visits in the same 90 day period that did not involve an overnight stay. For our analyses, the dependent variable was defined as an older adult who, in the 90 days prior to the follow-up assessment, had either a hospital stay or ED visit. Observation stays in the US data were coded as ED visits as they did not constitute a formal admission to the hospital.

### Analysis

Secondary analyses were carried out for older adults in the interRAI Home Care (HC) data set. Two assessments were used. Independent variables were drawn from the baseline assessment. The dependent measures, hospitalization and ED visits were drawn from the follow-up assessment – with the majority of the assessments completed between 2003 and 2008. A literature review was used to identify what others have found to be related to these outcomes. These factors helped to define the working list of independent variables. The strength of this approach was the availability of a broad set of variables, including clinical complications, disease diagnoses, treatments received, services used and functional and cognitive status.

The analyses proceed through a multi-step process. The first analysis assessed whether it is reasonable to assume that the same general risk factors are operational in each of the data cohorts. Can we pool the available cross-national data to create the risk model? This analysis is based on an odds ratio comparison of follow-up hospital/ED visits as driven by five common risk measures identified in the literature (see above). The measures are pneumonia, congestive heart failure, urinary tract infection, the absence of Alzheimers disease, and the number of medications taken (nine or more medications).

The first set of analyses in the model specification stage of the study, using the two-thirds derivation cohort, identified the baseline independent variables that do and do not predict the follow-up measure of hospitalization and ED use. Because of the enormous size of the sample, a wide variety of items (with very low measures of association) were expected to be significantly related at even very low levels (e.g., <.01) to the dependent measure.

The independent predictors tested fall into nine domains. Based on our review of the literature, the three major domains of interest are clinical complications (28 measures), disease diagnosis (19 measures), and specialized treatments (15 measures). The six other domains evaluated are cognition and communication (6 measures); mood and behavior (12 measures); social relations and informal supports (4 measures); functional status (11 measures); environmental conditions (10 measures); and services received (18 measures). Each of the identified independent measures was reviewed, and the best possible dichotomous form for the item specified. Univariate odds ratios (OR) were calculated next, and where the OR equaled or exceeded 1.30 as a risk factor or .75 or lower as a protective factor, the item was assumed to play a “substantive” role in understanding subsequent hospital and ED use. This identification step is based on all subjects in the derivation sample, pooled across the state, province, and country cohorts, with subsequent confirmation based on the re-test validation sample.

Stepwise logistic regression was used next to identify the characteristics that best identified elders who subsequently entered a hospital or visited an ED. These analyses were initiated in stages. In the first stage, we considered only diagnosis, clinical complexity, and specialized treatment measures, those with the most compelling face validity. After this pool had been reduced, including only measures in the multivariate model with an odds ratio of 1.2 in the derivation and validation samples, we then assessed how the measures in the six remaining domains might add to the model.

The items generated from the final multivariate logistic equation using the interRAI home care assessment measures comprise the interRAI Hospital-ED Risk Index.

## Results

**Figure 1:**
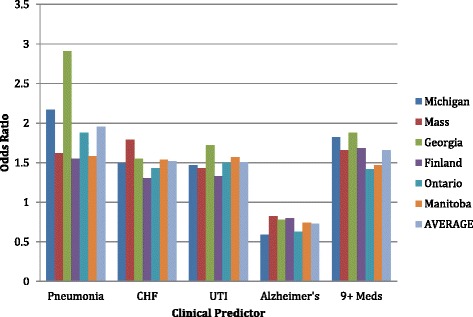
**Odds ratio of common clinical predictors of hospital-ED use across study cohorts.**

Figure 1 provides comparative, cross cohort, odds ratios when the follow-up hospital/ED visit measure is contrasted with five common risk measures identified in the literature. For each of the independent variables, the observed odds ratios follow a common pattern. Alzheimers is protective. The other four measures (pneumonia, congestive heart failure, urinary tract infection, and nine or more medications) each increase one’s risk of a hospital/ED visit. Thus, the remaining analyses in this paper are based on the use of the cross-national pooled dataset.

For the home care model derivation sample, the baseline hospital-ED visit rates (which reflect utilization in the 90 days prior to the independent variable measures) were significantly higher than the rates at the time of the follow-up assessment: hospital stay – 37.7% vs. 25.3%; ED – 19.8% vs. 15.7%. These data suggest that once the immediate crisis that brought the person into a home care program passed the hospital and ED utilization rate decline.

The follow-up hospitalization-ED rates for these elders receiving home care services also differed across countries. For the six population-based cohorts the rates are as follows: Massachusetts 27%; Michigan 29%; Finland 33%; Ontario 35%; Manitoba39%; and Georgia 41%. There are differences, but no country/state has an extremely low rate or high rate. This utilization phenomenon is common in all country datasets.

**Figure 2:**
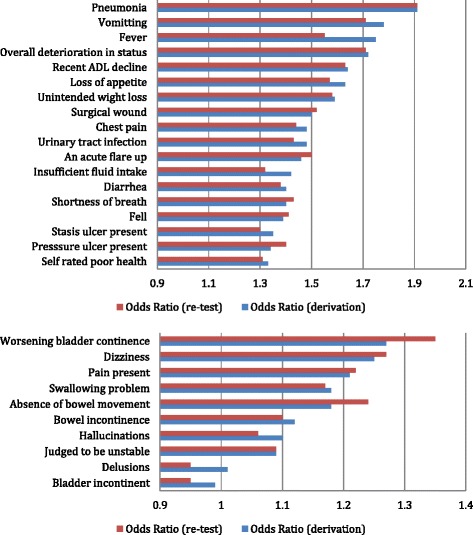
**Clinical complications compared to follow-up hospitalization/ED. Top – odds ratio greater than 1.3. Bottom – odds ratio less than 1.3.**

**Figure 3:**
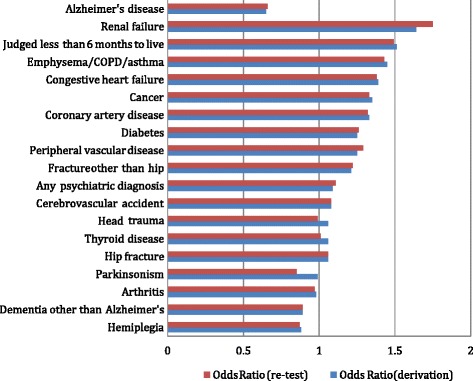
**Disease diagnosis -- odds ratios.**

**Figure 4:**
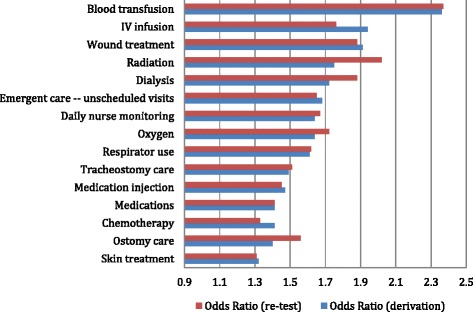
**Treatment odds ratios.**

Figures 2, 3 and 4 display an overview of how the independent measures in the three key domains of clinical complications, disease diagnoses, and specialized treatments relate to subsequent hospitalization or ED use. In the clinical complications domain (Figure 2), ten of the twenty-eight items have odds ratios less than 1.3, including the continence measures, pain, swallowing problem, hallucinations, and delusions. Among the eighteen clinical complication measures with higher, more meaningful odds ratios are pneumonia, urinary tract infection, fever, chest pain, diarrhea, unintended weight loss, a variety of skin conditions, and subject self reported poor health.

Figure 3 displays the odd ratios (with hospital and ED use) for nineteen disease diagnoses, of which the top seven in the figure meet or exceed our 1.3 odds ratio criterion required for the item to be presumed to have a meaningful relationship with hospital/ED use. Among these measures are coronary artery disease, congestive heart failure, cancer, emphysema, and renal failure. Also included in this list is the diagnosis that the person has six or fewer months to live. Finally, Alzheimers plays a protective role. Persons with this disease have a lower risk of subsequent hospital-ED use.

All fifteen specialized treatments meet our 1.3 or higher odds ratio criterion for having a meaningful relationship with hospital/Ed use (Figure 4). Although many are not in wide-scale use, when present persons receiving these complex treatments have an elevated risk of subsequent hospital-ED use. Of these measures, blood transfusion, IV infusion, wound treatment, radiation, and dialysis all have odds ratios that approach 2.0.

Of the eighteen items in the service domain, only one has a positive odds ratio in the 1.3 range or higher: alcohol or drug treatment program participation (1.44 OR/1.58 OR). Among services with lower odds ratios are all of the therapy services, medical oversight, and a variety of services in the home. Two of the services do play a protective role: day care (.66 OR/.67 OR) and home health aide (.73 OR/.73 OR).

For the items in the five remaining domains, none have odds ratios in the 1.3 or higher range. This list includes 12 mood and behavior items, 11 functional items, 10 environmental items, and six cognition-communication items.

**Figure 5:**
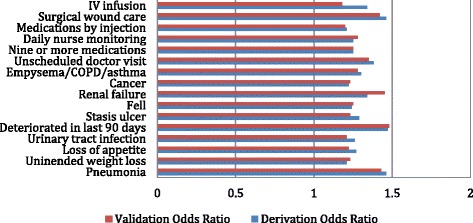
**Odds ratios of items in final multivariate logistic model.**

The final multivariate logistic model includes seven clinical complications, three disease diagnoses, and six treatments. Figure 5 presents the associated odds ratios for these sixteen items, with estimates provided for the derivation and validation samples (note, as there are no real differences between the two samples, this will be the last figure in this paper in which the validation results will be displayed.) In predicting follow-up hospital and ED use from baseline clinical complications, risk increases as the person accumulates an even longer list of clinical problems, specific disease diagnoses, and complex treatments. The list includes: infections (pneumonia and urinary tract infection), skin problems (stasis ulcers and wound care), recent deterioration (unintended weight loss, a major deterioration in status over the prior 90 days, unscheduled doctor visits, falls), the presence of serious disease (renal failure, emphysema, cancer), and close monitoring for non-trivial treatments (daily nurse monitoring, IV infusion, medication by injection).

It is rare for any one person to have all sixteen or even a majority of the sixteen risk factors. When this list of factors is summarized to create what we are calling the Hospital-ED Risk Index, only 4% of persons have a score of six or higher, while sixteen percent of persons have none of these risk factors.

**Figure 6:**
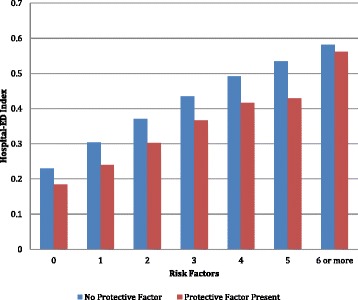
**Proportion of persons with hospital-ED use at follow-up across the categories of the hospital-ED risk index.**

The final specification of this risk model requires that we consider the three measures identified as playing a protective role: a diagnosis of Alzheimers disease, participation in day care, or visits by a home health aide. Two of these items entered the final multivariate model as risk factors: the presence of Alzheimers disease; participation in a day care program. Figure 6 displays the trend of increasing hospital/Ed use across the categories of the Hospital-ED Risk Index for those with and without these protective factors. On average, the presence of one or both of the protective factors translates into a one step drop in the average risk level for the person (about a 7% drop in risk).

**Figure 7:**
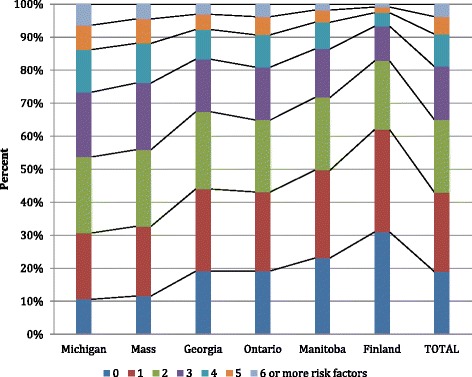
**Hospital-ED rIsk index distribution in total and by data cohort.**

Thus, in the final specification for the Hospital-ED Risk Index, we subtract one point from the total score for anyone who has one or both protective factors (Alzhimers, participation in day care). Figure 7 displays the distribution for the final Hospital-ED Risk Index in total and by data cohort. Two things of note: first, the proportion of persons with no risks differs by data cohort, going from about 10% in Michigan to 31% in Finland; and second, none of the data cohorts has more than 6% of the persons in the top risk category.

**Figure 8:**
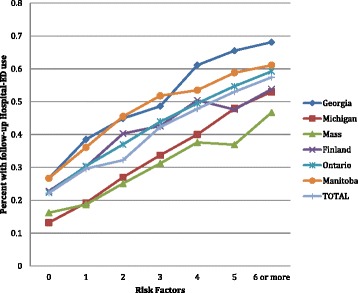
**Follow-up hospital ED use by hospital-ED risk index and cohort.**

Figure 8 displays the follow-up hospital-ED use proportions by cohort across the categories of the Hospital-ED Risk Index. The pattern is the same: as the risk count increases, use goes up. At the same time, there are differences in the rates by cohort, with persons in the Georgia cohort having somewhat higher rates and persons in the Massachusetts cohort having somewhat lower rates.

**Figure 9:**
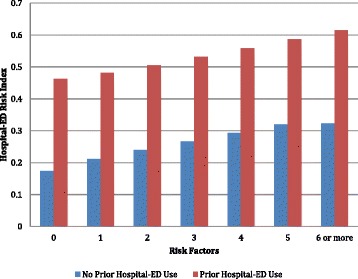
**Percent of persons with hospital-ED use at follow-up by the hospital-ED risk index and prior use.**

To understand better the nature of the risk model, Figure 9 displays the mean proportion of persons with hospital-ED utilization at follow-up controlled by both the Hospital-ED Risk Index and whether the person had a prior hospital-ED visit at the time of the baseline assessment. For those without a prior hospital-ED visit, the proportion with follow-up utilization rises steadily from 17.4% for those with zero risk factors to 32.3% for those with six or more risk factors. For those with prior hospital-ED use, the rate goes from 46.3% for those with no risk factors, to 61.5% for those with six or more risk factors. For both sub-groups the rates raise with the risk count. However, for those with prior hospital-ED use, the hospital-ED use at follow-up is higher in the no risk group than the rate observed for those without prior hospital-ED use who have six or more risk factors. Prior hospital-ED use is a powerful driver of subsequent behavior in and of itself. Given the differences in prospective hospital-ED use for those with and without prior utilization one could also create a summary interRAI Hospital-ED Risk Count Index where categories 0 to 6 represent persons with no prior use and categories 7 thru 12 represent persons with prior use.

**Figure 10:**
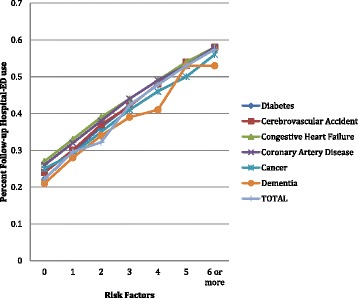
**Prospective hospital-ED rates by disease and hospital-Ed risk index score.**

Figure 10 displays the prospective hospital-ED rates by disease and Hospital-Ed Risk Index score. The risk model appears to be equally applicable across all disease diagnoses. There are no diseases in which the hospital-ED rates diverge from the expected pattern of increasing use across the Hospital-ED Risk Index. The clinical conditions, disease diagnosis, and treatment items making up the Hospital-ED Risk Index work equally well for each of the indicated diseases – be they cardiovascular, dementia, cancer, or diabetes.

## Discussion

The elderly represent only 13 percent of the population in the United States, but account for 37 percent of hospital discharges [33] and this phenomenon is not unique to the United States. Both to assure the highest quality of care and to control costs, one must understand why elders go to emergency departments and why they are admitted to the hospital at such a high rate. Certainly large numbers of re-hospitalizations are unnecessary. The Medicare Payment Advisory Commission in the US said that “potentially preventable” re-hospitalizations cost the American taxpayer $12 billion dollars a year [25]. As one manifestation of a renewed awareness of the need for change, in 2012, the US federal agency that pays for elder care, CMS, set new rules for readmissions for acute myocardial infarction, heart failure, and pneumonia and COPD [26]. As part of this process CMS has a formula for assessing the excess readmission ratio for a hospital, which, in turn, is affecting its reimbursement to hospitals.

What is the challenge? First, as Kansagara et al. [34] noted, while there are models that incorporate covariates, few models dig deeply into the clinical, functional, illness, and overall health status measures of elders. In our logistic equation, sixteen covariates enter the increasing risk of hospital and ED use model, and the measures are shown to be applicable to home care residents served in two Canadian provinces, three US states, and one European country. They also are shown to be relevant across a broad spectrum of disease categories. They represent a diverse set of clinical problems, including skin care issues, infections, weight loss and falls. In addition, they relate to a number of diagnostic conditions, including cancer, renal failure, and emphysema. Finally, a number of treatments also were found to increase use, including daily nurse monitoring, unscheduled doctor visits, receiving nine or more medications, and IV infusion. At the same time, a wide variety of measures did not enter the model – all of which failed to achieve a baseline OR of 1.3 or higher. These conditions do not seem to push subsequent utilization. By domain, the excluded measures that do not appear to play a large role follow. Cognition/communication: short-term memory, cognitive decision making, making self understood, understanding others; Mood/Depression indicators; Caregiver Distress; IADL Capacity; ADL Performance; Days Out/Physical Exercise; Bladder/Bowel Continence; a wide variety of disease diagnoses including hypertension, peripheral vascular disease, head trauma, hemiplegia, Parkinsons, arthritis, hip or other fracture, osteoporosis, cataract, glaucoma, diabetes, thyroid disease; and Clinical conditions: delusions, hallucinations, pain, unsteady gait, and dysphasia. Finally, the model includes two factors that are protective: no matter the number of clinical factors, somewhat lower use rates were observed for persons with Alzheimers disease and persons in a day care program.

The index created in this work both rests on earlier work and at the same time breaks new ground. It is based on earlier work in that it incorporates a series of measures used by others, including clinical complications such as pneumonia and urinary tract infection, and disease diagnoses such as cancer and emphysema [25,35-37]. At the same time we open new ground by first having considered a diverse group of clinical complications and diagnoses. We were able to focus on clinical factors that do and do not play a role in subsequent hospital-ED use. We also looked at how a variety of complex treatments relate to hospital-ED use, all were potential candidates and several entered our final model (e.g., IV infusion, 9 or medications, unscheduled doctor visits, and daily nurse monitoring). Finally, our work found that many types of items were not good candidates for the final model, including measures of function, cognition, communication, and mood.

The results from this secondary analysis are based on data generated from a comprehensive assessment of home care clients. As is the case with any analytical model, our approach was limited to the data measures available, in this case, the interRAI Home Care assessment. No clinical measures such as laboratory results, radiology, imaging and non-invasive scans were considered for this model and thus may be viewed as apparent limitations.

From a program perspective, persons who have been recently hospitalized are worthy of special comment. No matter the number of risk covariates, many will re-enter a hospital; and as risk factors increase so too does the likelihood of a readmission. At the same time, given the high re-hospitalization rate for those with none of the clinical risk factors in our model, one has to question the discharge process. What do we know about the transition of care? Were they discharged too early? Did they leave the hospital without an effective plan of care in the community? Could more have been done by the home care agencies to address the needs of these persons? Was there an appropriate degree of communication between the hospital staff and those caring for the person in the post-hospital setting? What did these production functions look like, was there a failure to see the full person as he/she passed through the care process?

As we look for ways to make appropriate changes to hospital-ED utilization, we should consider the possible use of incentives in a country. Although these may differ from nation to nation, it is now apparent that incentives play a major role in improving health care [38-40]. In the United States a number of factors may promote unnecessary use of the ED as well as admission to the hospital. For both financial and licensing reasons, skilled nursing facilities and home care agencies do not wish to provide care that might be judged to be acute. The reimbursement for medical care by a physician within a long term care facility is considerably below what could be billed for the same care at the acute care facility. Furthermore, the physician is reassured that it is far easier to manage an individual in the ED and the hospital because of the greater availability of technical, medical and nursing resources. As most elders and their families believe that a hospital is the best possible site of care, some segment of admissions and ED visits also may be pushed because of concerns about medical legal liability should such care not be offered.

## Conclusion

The Hospital ED Risk Index is produced each time the interRAI Home Care tool is used to complete an assessment of an elder enrolled in home care, While this index is complex with multiple predictive factors, the outcomes summary from the Home Care Tool provides a simple summary of this risk based on the number of risk factors and the number of protective factors present for any elder in home care. The assessor, elder, and possibly caregiver are provided with a score that reflects the risk for hospitalization or ER visit with an identification of the contributing factors. Knowledge of the risk factors present as well as any protective factors provided information to target interventions to address these factors and intervene as needed with the goal of preventing a future hospitalization or ER visit.

Looking to the future it is apparent that much care that has traditionally been provided in the hospital may be provided in the home setting. Leff and colleagues demonstrated clearly that large numbers of persons seen in an ED who were diagnosed with pneumonia, COPD, congestive heart failure and cellulitis could be managed at home for less cost and with shorter periods of acute illness [41]. The patients also had better satisfaction [42]. Using a hospital-risk index is a strategy that may be useful to reduce the number of unnecessary hospitalizations and ED visits.

It should also be noted that primary care physicians are becoming an ever smaller component of the medical profession, especially in the United States. Of note, the number of candidates entering the subspecialty of geriatric medicine, for example, is exceedingly small as compared to the number entering cardiology. In part this likely reflects the costs of education to obtain a medical degree and the marked difference in reimbursement for procedure-rich specialists compared with those who provide primary care. The limited number of geriatricians compounds the limited supply of primary care providers who may not have geriatric consultation available. In these instances, a Hospital ED Risk Index is a valuable resource.
